# Working memory capacity and redundant information processing efficiency

**DOI:** 10.3389/fpsyg.2015.00594

**Published:** 2015-05-27

**Authors:** Michael J. Endres, Joseph W. Houpt, Chris Donkin, Peter R. Finn

**Affiliations:** ^1^Department of Health, Behavioral Health AdministrationHonolulu, HI, USA; ^2^Department of Psychology, University of HawaiiHonolulu, HI, USA; ^3^Department of Psychology, Wright State UniversityDayton, OH, USA; ^4^School of Psychology, University of New South WalesSydney, NSW, Australia; ^5^Department of Psychological and Brain Sciences, Indiana UniversityBloomington, IN, USA

**Keywords:** working memory capacity, systems factorial technology, linear ballistic accumulator, individual differences, memory retrieval

## Abstract

Working memory capacity (WMC) is typically measured by the amount of task-relevant information an individual can keep in mind while resisting distraction or interference from task-irrelevant information. The current research investigated the extent to which differences in WMC were associated with performance on a novel redundant memory probes (RMP) task that systematically varied the amount of to-be-remembered (targets) and to-be-ignored (distractor) information. The RMP task was designed to both facilitate and inhibit working memory search processes, as evidenced by differences in accuracy, response time, and Linear Ballistic Accumulator (LBA) model estimates of information processing efficiency. Participants (*N* = 170) completed standard intelligence tests and dual-span WMC tasks, along with the RMP task. As expected, accuracy, response-time, and LBA model results indicated memory search and retrieval processes were facilitated under redundant-target conditions, but also inhibited under mixed target/distractor and redundant-distractor conditions. Repeated measures analyses also indicated that, while individuals classified as high (*n* = 85) and low (*n* = 85) WMC did not differ in the magnitude of redundancy effects, groups did differ in the efficiency of memory search and retrieval processes overall. Results suggest that redundant information reliably facilitates and inhibits the efficiency or speed of working memory search, and these effects are independent of more general limits and individual differences in the capacity or space of working memory.

## 1. Introduction

Working memory can be described as a multifaceted limited-capacity information processing system, comprising interrelated attention and memory subsystems that govern the controlled processing of goal-relevant information over short periods of time and in light of interference or distraction from goal-irrelevant information (Baddeley and Hitch, 1974; Baddeley, 1986, 2000; Baddeley and Logie, 1999). Complex or dual span tasks have been typically used to measure the processing “capacity” of working memory, quantifying the total “amount” of to-be-remembered information that can be accurately held in mind while resisting distraction from to-be-ignored information (Conway and Engle, 1994; Conway et al., 2005). Researchers have consistently shown dual span task performance decreases as a function of increases in to-be-remembered and ignored information, supporting the hypothesis that working memory is limited capacity in nature. Although this work has provided strong evidence that working memory capacity is limited, little is yet understood about the effect that redundant information has on working memory processing capacity and efficiency. The current research used an extreme groups approach and a novel redundant memory probes (RMP) task to investigate (a) the extent to which the “efficiency” or “speed” of working memory visual-search processes were affected by redundancies in target and distractor information, and (b) whether such redundancy effects depend on individual differences in “capacity” or “amount” of working memory resources. Here, a simplified linear ballistic accumulator (LBA) model (Brown and Heathcote, 2008; Donkin et al., 2009) of RMP task accuracy and response time was used to characterize working memory efficiency, while working memory capacity was characterized by performance on standard dual span tasks.

The redundant-target paradigm has been commonly used to investigate the efficiency or workload capacity of visual-search processes in divided-attention and short-term memory. In such experiments, participants are presented with stimuli containing 2, 1, or 0 target features. The participant's task is to decide whether or not stimuli contain at least 1 target feature as quickly and as accurately as possible. Redundancy gain effects are demonstrated by decreases in reaction time (RT) performance under redundant-target conditions relative to single-target conditions, indicating increases in the amount of target information facilitates processing efficiency or workload capacity (e.g., Townsend and Eidels, 2011) or potentially statistical facilitation (Raab, 1962). Conversely, increases in RT performance under no-target or distractor conditions relative to all others indicates that increases in the amount of distractor information inhibits processing efficiency or workload capacity (e.g., Townsend and Eidels, 2011), or potentially statistical inhibition (cf. Townsend and Wenger, 2004).

This work has shown redundant target information facilitates speed, and in some cases the accuracy, of visual-search processes while distractor identification is inhibited because it is defined based on the conjunction of multiple properties. Although redundancy effects have been reliably shown in tasks that index divided attention or short-term memory processes, little work has been done to characterize redundancy effects in tasks designed to measure working memory processes. The present research assumed that if working memory governs the interaction between divided attention and short-term memory processes, then tasks that tap both processes index more general working memory resources. Following from this assumption, it was hypothesized that redundant target and distractor information presented during short-term memory search would yield classic redundancy gain and loss effects on decision-making accuracy and RT that can be attributed to facilitation and inhibition of working memory information processing efficiency or workload capacity

Recently, Eidels et al. (2010) used an LBA model to quantify the efficiency and workload capacity of cognitive processes underlying redundant-target effects in a divided-attention experiment. Results showed that the LBA model was sensitive to the redundancy gain effects observed for choice accuracy and RT, such that model estimates of internal evidence accumulation or drift-rates showed greater efficiency in divided attention under redundant-target conditions relative to single-target conditions. Model simulations of participant drift-rate data also predicted individual differences in workload capacity as indicated by Townsend and colleagues' capacity coefficient (e.g., Townsend and Nozawa, 1995; Townsend and Wenger, 2004; Houpt and Townsend, 2012; Burns et al., 2013; Houpt et al., 2014) which characterized participant's divided attention as super, unlimited, or limited capacity. Crucially, results showed participants with larger differences between redundant-target and single-target drift-rates showed super capacity in divided attention, whereby redundant targets facilitated or increased the workload capacity of target recognition. In contrast, participants with smaller drift-rate differences tended to show limited capacity in divided attention, whereby redundant targets inhibited or decreased the workload capacity of target recognition. In sum, drift-rate efficiency and workload capacity measures showed convergent evidence that suggested individuals can differ in the magnitude of redundancy gain effects on divided attention, whereby some individuals show facilitation in processing efficiency, and others experience inhibition. The present research builds from this work by using the LBA model to (a) investigate redundancy gain and loss effects using a novel working memory experiment, and (b) determine the extent to which such effects differ between individuals classified as having low or high working memory capacity on dual span tasks.

In our current work, we deviate from the (Eidels et al., 2010) approach by using the average of the single conditions processing rates as the baseline for comparison to the dual conditions. The advantage to our approach was that it did not require additional complexity and model development beyond the standard LBA. The disadvantage of our approach compared to the Eidels et al. approach is that the baseline model does not match the traditional unlimited-capacity, independent parallel model baseline (cf. Townsend and Nozawa, 1995; Houpt et al., 2014); instead, our baseline is essentially a fixed-capacity coactive model. A fixed-capacity coactive model predicts the processing rates in the dual conditions will be the sum of one half the processing rates in the single conditions because in that model information regarding target presence or absence is summed across the two sources, but each process is only half as efficient due to spreading a fixed amount of resources across the sources (cf. Houpt and Townsend, 2011). While we do not have a strong argument for a fixed-capacity coactive baseline over an unlimited-capacity parallel model, our focus is not to determine whether individual participants exhibit super, unlimited, or limited workload capacity in the RMP task. Rather, our focus is on the extent to which redundancy effects in the RMP task vary as a function of individual differences in performance on other well-established working memory span tasks. This focus minimizes the issue of specifying a baseline model because redundancy effects are operationalized experimentally, as given by the magnitude of differences between performance indicators obtained under redundancy and singleton conditions.

**Figure 1:**
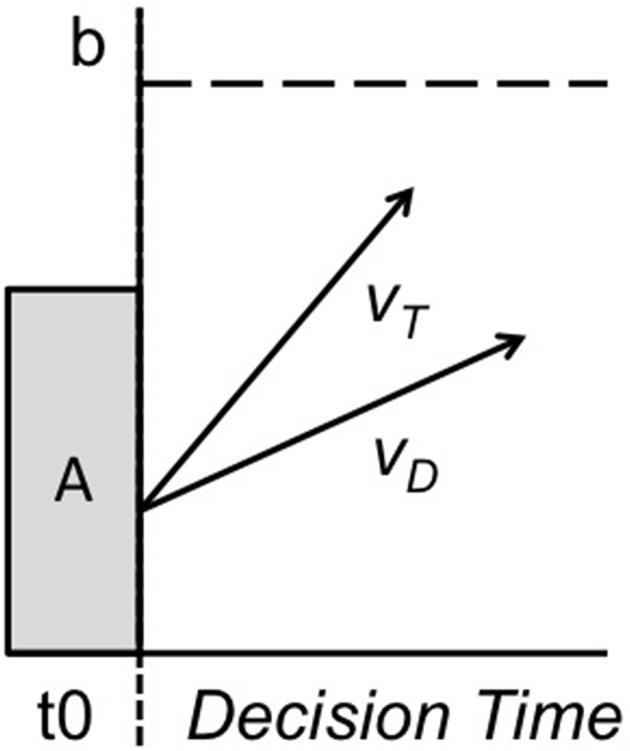
**Linear ballistic accumulator (LBA) model of working memory search and decision- making process assuming an underlying coactive mental architecture**. On any given trial, this LBA unit governs the time taken to execute a target (T) or distractor (D) response in the presence of some memory probe stimulus. Working memory search and decision-making process begins and ends with some non-decision time (*t*_0_) related to sensory input and motor response output. A decision is determined by the rate at which evidence accumulates for target (VT) and distractor (VD), with drift-rates initiating from some starting point (A) and racing one another toward some threshold for sufficient evidence (b). Whichever drift-rate crosses threshold first governs the response. Evidence accumulates according to a standard normal distribution with mean 0 and unit variance.

As in Figure 1, the current LBA model had 5 parameters (*t*_0_, *A*, *b*, *v*, and *s* = 1) that were assumed to govern the process of scanning short-term memory and deciding whether a given memory probe contained target (match) or distractor (non-match) information. Although alternative sequential sampling models are capable of characterizing RMP task performance (e.g., Ratcliff, 1978), these models tend to lead to similar conclusions (Donkin et al., 2011). The current LBA model used full RT distributions for correct and incorrect choices to estimate the rate at which evidence for target and distractor responses accumulate during the memory search process. A decision is made whenever the first accumulation process reaches an internal threshold criterion for sufficient evidence. In Figure 1, the *b* parameter represents the threshold of sufficient evidence for a response. High *b*-values reflect a preference for more information before making a decision. The *A* parameter represents the amount of evidence in each accumulator at the beginning of the trial. Higher values of *A* reflect a preference for responding fast. The *t*_0_ parameter represents elements of the RT distribution that are not associated with the decision-making process, such as perceptual encoding or motor execution latencies. Higher values of *t*_0_ reflect slower perceptual encoding and response execution. The *v* parameter represents the average rate of evidence accumulation for either the target (*v_T_*) or distractor (*v_D_*). High values of *v* reflect steeper or faster rates of evidence accumulation. The *s* parameter represents the standard deviation of the *v* parameter estimate, and is set to 1. Here, an accuracy adjusted drift rate, denoted (*V*), operationalized the process of accumulating accurate evidence for target and distractor decisions. The *V* measure was calculated by subtracting *v* obtained on incorrect trials from *v* on correct trials (*V* = *v*_correct_ − *v*_incorrect_).

In terms of LBA parameters, our baseline prediction was formalized as *V*_RedundantProbe_ = 0.5 (*V*_SingleProbe1_ + *V*_SingleProbe2_). Specifically, redundancy effects were evaluated as the inequality resulting from contrasting *V* obtained under redundancy conditions vs. the V obtained under singleton conditions, e.g., *V*_RedundantTarget_ versus 0.5 (V_ColorTarget_ + *V*_LetterTarget_). Note that using a single information accumulator to represent information accumulation for the redundant probe trials, and assuming that drift rate is a linear combination of the drift rate of the single probe processes, implies a coactive (i.e., information pooling) process. The “fixed-capacity” comes from the fact that we scale the sum by 0.5, or one over the number of information sources, when we take the average of the single probe drift rates.

The LBA model output *t*_0_, *A*, and *b* parameter values, along with 10 separate drift-rates, reflecting correct (*v*_correct_) and incorrect (*v*_incorrect_) evidence accumulation rates over each of the memory probe conditions (RT, ST, TD, RD, ST). Five accuracy adjusted drift-rates (*V*) were then derived by subtracting *v*_incorrect_ from *v*_correct_ for each condition separately, yielding the *V*_RT_, *V*_ST_, *V*_TD_, *V*_RD_, and *V*_SD_ values.

**Figure 2:**
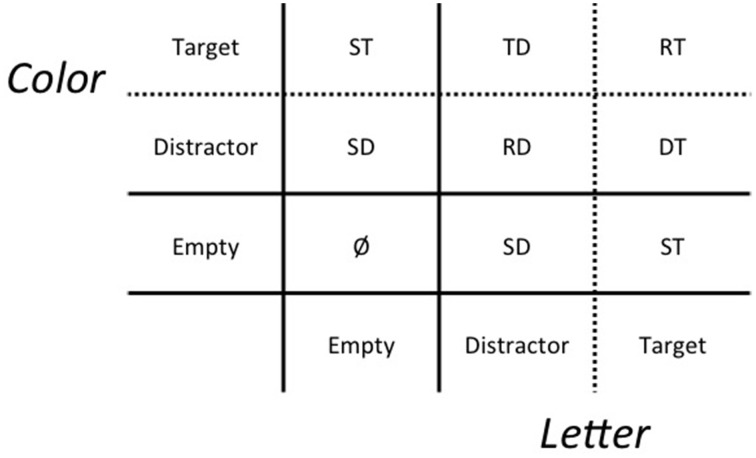
**Double-factorial redundant memory probes task factor 2 manipulation of target and distractor memory probe redundancy**. Memory probe stimuli vary in the amount of to-be- remembered (target) or to-be-ignored (distractor) color and letter features. RT, redundant target; TD, target and distractor; DT, distractor and target; RD, redundant distractor; ST, single target; SD, single distractor. For simplicity, TD and DT were combined to form a single two-dimensional target/distractor TD condition, and one-dimensional color and letter stimuli were combined to form separate SD and ST conditions.

The present research investigated two main aims. The first was to examine the effects of redundancy on performance in a novel task designed to study the interaction between divided-attention and short- term memory processes in working memory, which we call the redundant memory probes (RMP) task. Illustrated in Figure 2, and described in greater detailed later, the RMP task systematically varied the amount of to-be-remembered (target) and to-be-ignored (distractor) information present during short-term memory search. Consistent with previous research, choice accuracy, mean response time (mRT), and LBA model drift-rate measures were used to quantify redundancy effects in the RMP task. Based on previous research, it was hypothesized that a redundant-target (RT) condition would yield higher accuracy, faster mean reaction time (mRT), and larger LBA model drift-rates when contrasted against single-target (ST) conditions (*V*_RT_ > *V*_ST_). A redundant-distractor condition also was hypothesized to yield lower accuracy, slower mRT, and smaller drift-rates when contrasted against the single-distractor (SD) condition (*V*_RD_ < *V*_SD_). Mixed-target and distractor (TD and DT) conditions also were included to investigate the effects of overlapping target-distractor information on choice accuracy, mRT, and drift-rates, although we did not have any a priori predictions about the ordering of those drift rates relative to the other trial types (*V*_TD_, *V*_DT_?*V*_ST_).

The second aim was to examine whether individuals classified as having high or low working memory capacity (WMC), as determined by performance on traditional dual span tasks, differed in the magnitude of redundancy gain and loss effects on the RMP task. This extreme groups approach was used to determine whether individuals who are known to differ on well-established measures of WMC also differ with regard to their sensitivity to redundancy gain and loss effects and overall efficiency in working memory visual search. Based on previous working memory individual differences research, it was hypothesized that individuals with low WMC would show lower accuracy, slower mRT, smaller drift-rates, and be more susceptible to distractor information while processing target information than those with high WMC. We also expected to find an interaction between experimentally driven redundancy effects and WMC individual differences. Specifically, we hypothesized that the magnitude of redundancy effects would depend on WMC individual differences, such that individuals with low WMC would show less redundancy gain and loss effects.

## 2. Materials and methods

### 2.1. Participants

#### 2.1.1. Sample characteristics

The sample consisted of 170 young adults (96 men, 74 women; χ^2^ = 2.85, *p* > 0.05) ranging in age from 18 to 30 (mean = 20.89 ± 2.31). The sample was 77% White, 8% African American, 6% Asian, Indian, or Middle Eastern, 6% Hispanic or Latino, and 3% multiple ethnicities. Men were older than women [*t*_(168)_ = 1.96, *p* < 0.05]. However, gender was not associated with differences on any other study variable.

#### 2.1.2. Study recruitment

Participants were recruited from a subject pool of participants who completed a larger study on the personality, cognitive, and decision making correlates of substance use and antisocial behavior problems in young adults. Participants in the larger study were recruited using advertisements posted around the campus and surrounding community of a large Midwestern university. Advertisements were also placed in local and student newspapers. Advertisements were designed to attract individuals with varying degrees of lifetime problems with substance use and impulse control. This approach has been effective in attracting responses from individuals who vary in performance on cognitive tasks assessing intelligence, associative learning, short-term memory, working memory, and approach-avoidance decision making (Finn et al., 2002, 2009; Endres et al., 2011, 2014).

Advertisement respondents were telephone screened for inclusion criteria of being between 18 and 30 years of age, able read/speak English, at least 6th grade education, and without a history of psychosis or head trauma. On the day of testing subjects were further screened to ensure participants did not use alcohol or drugs in the past 12 hours, were not experiencing symptoms of withdrawal or fatigue, and had a breath alcohol content of 0.0%.

Participants in the current sub-study were recruited based on a stratified random sample of main study participants (*N* = 507). Participants who completed the entire main study protocol were categorized as having low, moderate, or high histories of substance use and antisocial behavior based on an unsupervised cluster analysis of participant self-reported history with alcohol, drugs, childhood conduct problems, and adult antisocial behavior. A total of 180 participants (60 from each of the three groups) were solicited for participation in the present study with a final response rate of 94.44%. Based on previous research noting a negative association between executive cognitive functioning (e.g., intelligence, associative learning, and working memory) and individual's history of substance use and antisocial behavior (Finn et al., 2009), participants in the current stratified sample also were expected to vary greatly with respect to working memory and executive decision-making ability.

#### 2.1.3. Dual span tasks

Working memory capacity (WMC) was assessed using two different complex-span tests, the Operation-Word Span test (OW; Conway and Engle, 1994) and a modified version of the Auditory Consonant Trigram test (AC; Brown, 1958; Finn et al., 2009; Endres et al., 2011). These tasks operationalize WMC as the total number of primary memory items that can be correctly recalled after performing a second unrelated cognitive task. The OW test was experimenter based and assessed the total number of words that were correctly recalled after performing a mathematical operation. For example, participants were asked to determine whether a mathematical operation was correct and presented with a word to-be-remembered (2 × 5 = 12? DOG). After a series of operation-word trials, participants were asked to recall the words in there correct order of presentation in the series. The AC test also was experimenter based and assessed the total number of consonant letters, from a string of letters (e.g., r, d, t, and l), that could be remembered after counting backwards by 3's from a random three-digit number (e.g., 379) for a pre-determined length of time (e.g., 18 or 36 s). Several studies indicated that the OW and AC tests are valid indicators of the limited capacity nature of working memory, wherein accuracy decreases as a function of increases in primary memory items and secondary cognitive loads (Engle et al., 1999; Endres et al., 2011). Consistent with previous research, a composite WMC factor score was created by estimating the covariance among the total number of items correctly recalled on the OW and AC tasks using maximum likelihood extraction (Engle et al., 1999; Finn et al., 2009; Endres et al., 2011). This WMC factor score variable was eventually dichotomized to reflect individual differences in high and low WMC in repeated measure analyses. Individuals were classified as having low or high WMC based on a median split (median = 0.03) of maximum likelihood estimated WMC factor scores (Cronbach's Alpha = 0.67, mean = 0, *SD* = 0.88, skew = −0.34, kurtosis = −0.36).

#### 2.1.4. Redundant memory probe tasks

The redundant memory probes (RMP) task was designed to study the interaction between divided- attention and short-term memory processes in working memory. The task used basic study-test (Sternberg, 1966) and varied response mapping (Schnieder and Shiffrin, 1977) procedures embedded within a double-factorial design Townsend and Wenger (2004) to examine the effects of redundant target and distractor information on the processes of searching short-term memory for color and letter information.

**Figure 3:**
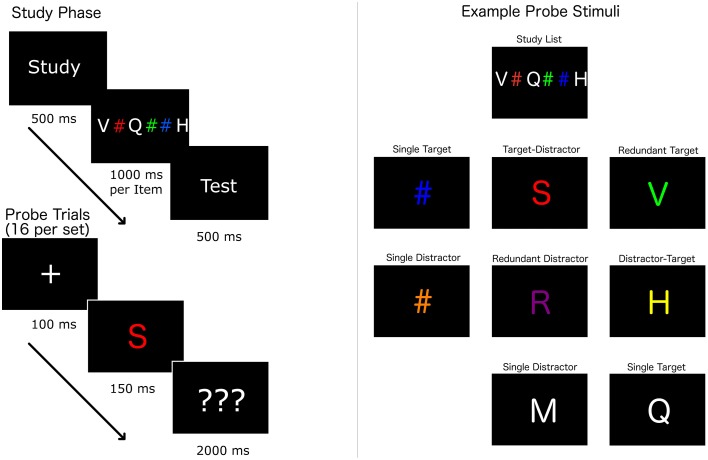
**Redundant memory probe (RMP) task example of a block with a 6 item (3 color and 3 letter) memory list and potential memory probes**. The left side indicates the task flow within a block. The participants are first exposed to a study list for 1000 ms per item in the list, then the test phase begins. The test phase consists of 16 trials where the probe on each trial is one of the types indicated on the right side. Redundant target probes are letters from the study list with one of the study list colors. Target-distractor trials contain a color from the study list but a letter that was not on the list. Distractor-target trials contain a letter from the study list but a color that was not on the list. Redundant distractor trials have a letter and a color that were both not on the study list. Single color targets were a hash mark with a color from the list. Single letter targets were a letter from the list in white. Single color distractors were colored hash marks with colors that were not on the list. Single letter distractors trials were white letters that were not on the list.

The study-test procedure (Figure 3) involved the initial rehearsal of memory lists varying in length and composition of color and letter items (Factor 1), followed by the serially matching of 16 memory-test probes with and without redundant target and distractor features (Factor 2). During the study phase, participants rehearsed memory lists containing either 1 or 3 color items and 1 or three letter items for a period of time lasting 1 s per memory list item. Memory lists were 2, 4, or 6 items in length, and there were 4 list types (1-color/1-letter, 1-color/3-letter, 3-color/1-letter, and 3- color/3-letter) each with 6 different memory sets, totaling 24 lists in the task.

During the test phase, participants were briefly shown memory-test probes. Each probe was a single character. Probes that were colored (non-white) letters are referred to as dual probes. Probes that were either a white letter or a colored hash symbol are referred to as single probes. Probes could have 0, 1, or 2 target or distractor features. There were 8 probe types (Figure 2): redundant dual targets (RT) or distractors (RD), mixed color and letter dual targets and distractors (TD and DT), single color or letter targets (ST), and single color or letter distractors (SD).

Note that the participants were asked to say yes if either the color or letter of the probe was in the memory set. Hence, the dual probes to which the participants should have responded no (distractors) were defined by the conjunction of the color being outside of the memory set and the letter being outside of the memory set. The probes for which both color and letter were in the memory set had redundant target information. Memory test probes representing targets in a given study-test procedure could be distractors in other study-test sets (varied response mapping procedure), which was assumed to generate proactive interference.

#### 2.1.5. Dependent measures

Consistent with previous research, choice accuracy, mRT, and LBA model drift-rate estimates, which incorporates both accuracy and RT information, were used to investigate redundancy effects on test- phase performance by contrasting RT and RD with ST and SD, respectively. Performance estimates were aggregated across Factor 1, study set size, because memory probe redundancies were manipulated during the test phase (Factor 2). As in Figure 2, performance estimates also were aggregated across the mixed TD and DT, as well as single target (ST) and single distractor (SD) test probe types, because the task was designed so that: (a) color and letter elements had equal a priori stimulus presentation probabilities across the 24 study lists and 8 test probe types, and (b) target- distractor discriminability was held constant for the different color and letter elements of study lists and test probes.

### 2.2. Data analyses

Separate 2 × 2 repeated measures ANOVAs were used to examine the within-subjects effects of redundant information on RMP task performance measures as a function of between-subjects differences in WMC on dual span tasks. Based on previous research, the within-subjects factor in repeated measures analyses reflected planned comparisons for redundancy gain (RT vs. ST conditions), loss (RD vs. SD), and mixed (TD vs. ST) effects. Planned comparisons were conducted separately for gain, loss and mixed effects. Based on subject recruitment, the dichotomized (median split) WMC factor score variable was used as the between-subjects factor in all repeated measures analyses. Analyses were conducted separately for choice accuracy (percent correct), mRT (on correct trials), and accuracy adjusted LBA drift-rate performance measures. Within-subjects and between- subjects effect sizes were examined with partial eta-square estimates.

## 3. Results

### 3.1. Descriptive statistics

The low (*n* = 85) and high (*n* = 85) WMC groups did not differ in gender composition (χ^2^ = 2.16, *p* > 0.05) or average age [*t*_(168)_ = 1.06, *p* > 0.05]. However, groups did differ in average IQ [*t*_(167)_ = − 3.66, *p* < 0.001] and years of education [*t*_(168)_ = −3.66, *p* < 0.001].

### 3.2. Individual LBA model fits

**Figure 4:**
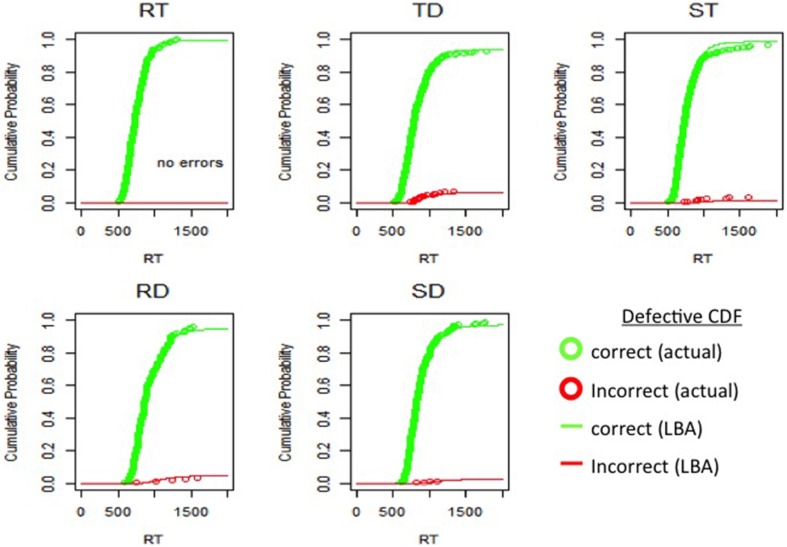
**Example subject's defective cumulative density functions illustrating the probability of observing correct (green font) and incorrect (red font) responses on or before some response time (RT)**. Subject's actual (open circles) data LBA simulated (lines) data plotted against each other for RT, redundant target; TD, mixed target/distractor; RD, redundant distractor; ST, single target; SD, single distractor conditions.

Model fit was examined by using subject's LBA model parameters to simulate accuracy and RT data, and then comparing these simulations to subject's actual accuracy and RT data. For example, Figure 4 shows one subject's LBA model simulated defective cumulative density functions (CDF) plotted against that subject's actual defective CDFs. In Figure 2, LBA model simulated CDFs for correct and incorrect responses in RT, TD, ST, RD, and SD test-probe conditions showed consistent overlap with actual CDFs collected in these respective conditions. The mean parameter value and standard deviation across participants is shown in Table 1.

**Table 1:** **Accuracy adjusted drift-rates by redundancy condition and percentile grouping**.

	**Low 20% (*n* = 34)**		**High 80% (*n* = 34)**	
**Condition**	**Mean**	**(*SD*)**	**Mean**	**(*SD*)**
Non-decision time (*t*_0_)	64.34	50.24	73.56	65.61
Starting point (*A*)	7.35	1.33	7.22	1.25
Threshold criterion (*b*)	8.67	0.25	8.58	0.22
Redundant target (*V*_RT_)	3.00	0.94	3.68	1.32
Single target (*V*_ST_)	2.63	0.74	3.09	0.63
Target and distractor (*V*_TD_)	1.96	0.93	2.33	0.83
Redundant distractor (*V*_RD_)	2.19	0.71	2.37	0.62
Single distractor (*V*_SD_)	2.25	0.73	2.80	0.82
	**Low 50% (*n* = 85)**		**High 50% (*n* = 85)**	
Non-decision time (*t*_0_)	67.12	48.38	73.01	65.40
Starting point (*A*)	7.33	1.28	7.30	1.22
Threshold criterion (*b*)	8.65	0.23	8.60	0.22
Redundant target (*V*_RT_)	3.30	1.37	3.66	1.34
Single target (*V*_ST_)	2.86	0.78	3.11	0.64
Target and distractor (*V*_TD_)	1.95	1.08	2.28	0.89
Redundant distractor (*V*_RD_)	2.24	0.80	2.43	0.69
Single distractor (*V*_SD_)	2.39	0.87	2.69	0.71

### 3.3. Effects of WMC on LBA model non-decision time, starting point, and threshold

No WMC group differences were found for LBA model parameters *t*_0_ [*t*_(168)_ = 0.67, *p* > 0.05], *A* [*t*_(168)_ = −0.16, *p* > 0.05], or *b* [*t*_(168)_ = −1.36, *p* > 0.05]. For the High EMW capacity group, mean non-decision time, starting point, and threshold were 73.01±65.4, 7.30±1.28 and 8.66±0.22 respectively. For the low EMW capacity group, mean non-decision time, starting point, and threshold were 67.12±48.38, 7.33±1.28, and 8.65±0.23 respectively. These results suggest WMC individual differences are not involved in RMP task decision-making processes related to early perceptual coding and later response execution latencies, nor setting preferences for response types or sufficient evidence for responding.

### 3.4. Effects of redundant target information and WMC on RMP task performance

#### 3.4.1. Accuracy

**Figure 5:**
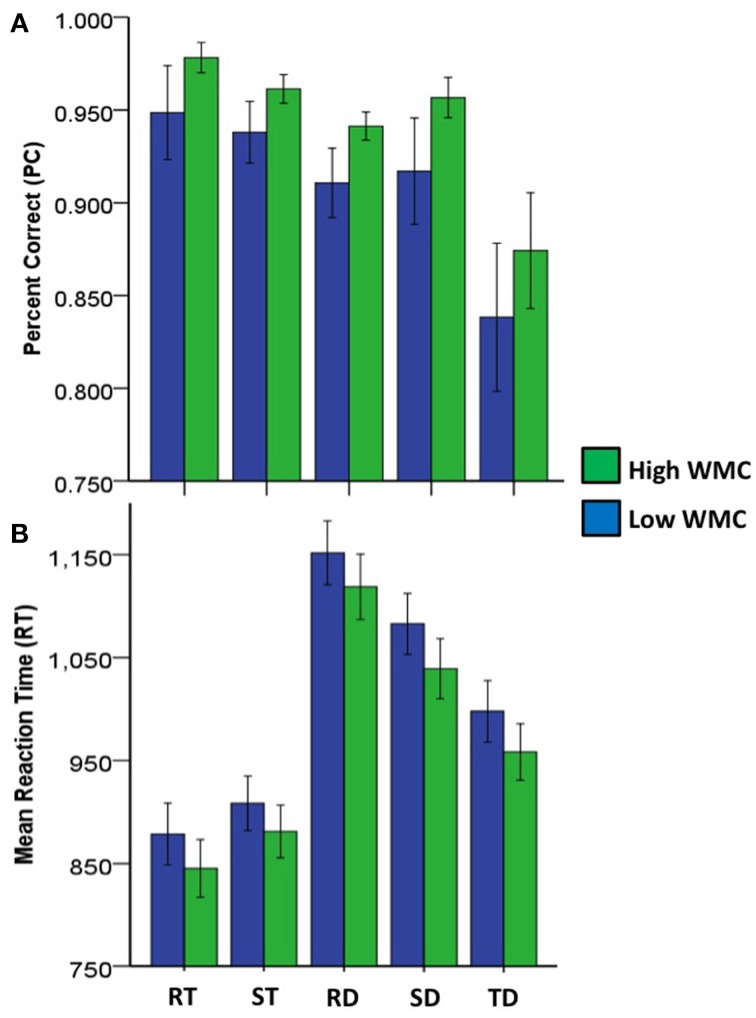
**Bar graphs with 95% confidence intervals for mean accuracy (A) and response time (B) by redundancy condition and working memory capacity (WMC) groupings**. RT, redundant target; TD, target and distractor; DT, distractor and target; RD, redundant distractor; ST, single target; SD, single distractor.

Figure 5A, hit rates were facilitated by redundant-target information. These effects did not depend on WMC differences, even though those with high WMC were generally better at recognizing targets than those with low WMC. Within subjects tests showed target percent correct (PC) was higher for redundant color and letter targets, relative to single color targets or single letter targets [RT > ST, *F*_(168)_ = 7.14, *p* < 0.01, partial η^2^ = 0.04]. Between subjects tests showed those classified as high WMC had higher overall target PC than those classified as low WMC [*F*_(168)_ = 6.67, *p* < 0.01, η_2_ = 0.04]. No interaction between redundant targets and WMC differences was found for target PC [*F*_(168)_ = 0.38, *p* > 0.05, η2 < 0.01].

#### 3.4.2. Correct trials mRT

Figure 5B, shows mRT on for hits were facilitated by redundant target information, and these effects did not depend on WMC differences. Although those with high WMC tended to be faster at recognizing targets than those with low WMC, these differences did not reach statistical significance.

Within subjects tests showed mRT was shorter for redundant color and letter targets, relative to single color targets or single letter targets [RT < ST, *F*_(168)_ = 116.65, *p* < 0.001, partial η^2^ = 0.41]. Between subjects tests showed those classified as high WMC did not differ in mRT from those classified as low WMC in overall mRT for targets [*F*_(168)_ = 2.46, *p* > 0.05, partial η^2^ = 0.01]. No interaction between redundant targets and WMC differences was found for mRT [*F*_(168)_ = 0.99, *p* > 0.05, partial η^2^ = 0.01].

#### 3.4.3. LBA drift-rates

**Figure 6:**
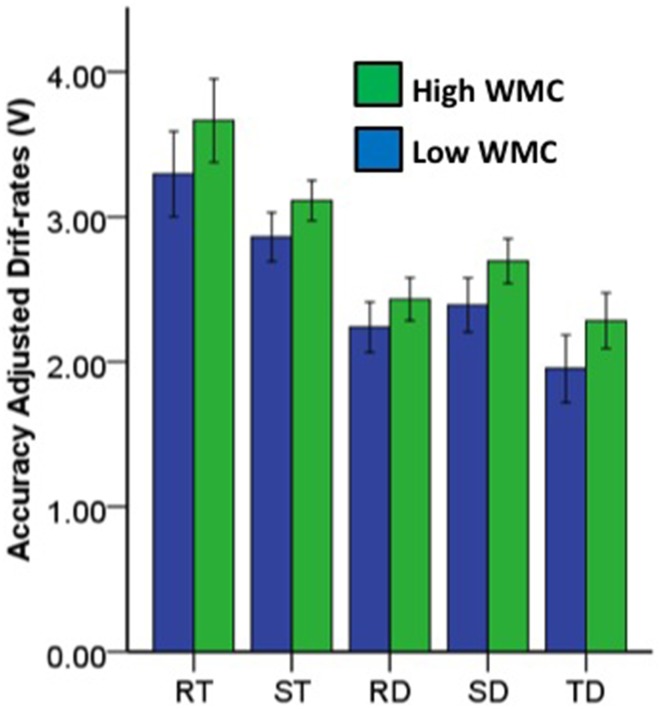
**Bar graphs with 95% confidence intervals for mean LBA model accuracy adjusted drift-rates by redundancy condition and working memory capacity (WMC) groupings**. RT, redundant target; TD, target and distractor; DT, distractor and target; RD, redundant distractor; ST, single target; SD, single distractor.

Figure 6 shows accuracy adjusted drift-rates (*V*) were facilitated by redundant-target information; and, these effects did not depend on WMC differences, even though those with high WMC were generally more efficient in target recognition than those with low WMC. Within subjects tests showed *V* was larger for redundant color and letter targets, relative to single color targets or single letter targets [*V*_RT_ > *V*_ST_, *F*_(168)_ = 25.03, *p* < 0.001, partial η^2^ = 0.13]. Between subjects tests showed those classified as high WMC had larger overall *V* for targets than those classified as low WMC [*F*_(168)_ = 5.41, *p* < 0.05, partial η^2^ = 0.03]. No interaction between redundant targets and WMC differences was found for *V* [*F*_(168)_ = 0.36, *p* > 0.05, partial η^2^ < 0.019].

### 3.5. Effects of redundant distractor information and WMC on RMP task performance

#### 3.5.1. Accuracy

Figure 5A, shows redundant-distractor information had an inhibitory effect on correct rejection rates, but these effects did not reach statistical significance. However, those with high WMC were generally better at recognizing distractors than those with low WMC. Within subjects tests showed PC for redundant color and letter distractors was not significantly different from PC for single color distractors or single letter distractors [RT = ST, *F*_(168)_ = 3.27, *p* > 0.05, partial η^2^ = 0.02]. Between subjects tests showed those classified as high WMC had higher distractor PC than those classified as low WMC [*F*_(168)_ = 9.25, *p* < 0.01, partial η^2^ = 0.05]. No interaction between conjunctive distractors and WMC differences was found for PC [*F*_(168)_ = 0.57, *p* > 0.05, partial η^2^ < 0.01].

#### 3.5.2. Correct trials mRT

Figure 5B, shows mRT on correct trials was inhibited for redundant distractors, and these effects did not depend on WMC differences. Those with high WMC were generally faster at recognizing distractors than those with low WMC, but these effects did not reach statistical significance. Within subjects tests showed mRT was longer for redundant color and letter distractors, relative to single color distractors or single letter distractors [RD > SD, *F*_(168)_ = 273.75, *p* < 0.001, partial η^2^ = 0.62]. Between subjects tests showed those classified as high WMC did not differ from those classified as low WMC in distractor mRT [*F*_(168)_ = 3.26, *p* > 0.05, η^2^ = 0.02]. No interaction between conjunctive distractors and WMC differences was found for mRT [*F*_(168)_ = 3.26, *p* > 0.05, partial η^2^ < 0.01].

#### 3.5.3. LBA drift-rates

Figure 6 shows accuracy adjusted drift-rates (*V*) reduced for redundant-distractor information. These effects did not depend on WMC differences, even though those with high WMC were generally more efficient at recognizing distractors than those with low WMC. Within subjects tests showed *V* was smaller for redundant color and letter distractors, relative to single color distractors or single letter distractors [*V*_RD_ < *V*_SD_, *F*_(168)_ = 9.86, *p* < 0.01, partial η^2^ = 0.06]. Between subjects tests showed those classified as high WMC had larger overall *V* for distractors than those classified as low WMC [*F*_(168)_ = 6.40, *p* < 0.05, partial η^2^ = 0.04]. No interaction between conjunctive distractors and WMC differences was found for *V* [*F*_(168)_ = 0.69, *p* > 0.05, partial η_2_ < 0.01].

### 3.6. Effects of mixed target/distractor information and WMC on RMP task performance

#### 3.6.1. Accuracy

Figure 5A, shows mixed target-distractor information had an inhibitory effect on hit rates, and these effects did not depend on WMC differences. Those with high WMC were better at recognizing targets while ignoring distractors than those with low WMC, but these effects did not reach statistical significance. Within subjects tests showed PC was lower for mixed color and letter targets and distractors, relative to single color targets or single letter targets [TD < ST, *F*_(168)_ = 76.32, *p* < 0.001, partial η^2^ = 0.31]. Between subjects tests showed those classified as high WMC did not significantly differ from those classified as low WMC in PC for mixed color and letter targets and distractors [*F*_(168)_ = 3.47, *p* > 0.05, η^2^ = 0.02]. No interaction between mixed color and letter targets and distractors and WMC differences was found for PC [*F*_(168)_ = 0.34, *p* > 0.05, partial η^2^ < 0.01].

#### 3.6.2. Correct trials mRT

Figure 5B, shows mRT on correct trials was inhibited by mixed target-distractor information, and these effects did not depend on WMC differences. Those with high WMC were generally faster at recognizing targets while ignoring distractors than those with low WMC, but these effects did not reach statistical significance. Within subjects tests showed mRT was longer for mixed color and letter targets and distractors, relative to single color targets or single letter targets [TD > ST, *F*_(168)_ = 513.49, *p* < 0.001, partial η^2^ = 0.75]. Between subjects tests showed those classified as high WMC did not differ from those classified as low WMC in mRT for mixed color and letter targets and distractors [*F*_(168)_ = 3.05, *p* > 0.05, η^2^ = 0.02]. No interaction between mixed color and letter targets and distractors and WMC differences was found for mRT [*F*_(168)_ = 2.74, *p* > 0.05, η^2^ = 0.02].

#### 3.6.3. LBA drift-rates

Figure 6 shows accuracy adjusted drift-rates (*V*) were inhibited by mixed target-distractor information. These effects did not depend on WMC differences, even though those with high WMC were generally more efficient at recognizing targets while ignoring distractors than those with low WMC. Within subjects tests showed *V* was smaller for mixed color and letter targets and distractors, relative to single color targets or single letter targets [*V*_TD_ < *V*_ST_, *F*_(168)_ = 175.79, *p* < 0.001, partial η^2^ = 0.51]. Between subjects tests showed those classified as high WMC had larger *V* for mixed color and letter targets and distractors than those classified as low WMC [*F*_(168)_ = 6.38, *p* < 0.05, partial η^2^ = 0.04]. No interaction between mixed color and letter targets and distractors and WMC differences was found for *V* [*F*_(168)_ = 0.37, *p* > 0.05, partial η^2^ < 0.01].

### 3.7. Additional analyses

To examine the stability of our findings, we conducted supplemental analyses using a more extreme percentile grouping criterion for dual span task WMC factor scores than a median split. As shown in Table 1, for adjusted drift rates, the direction and pattern of repeated measures effects did not differ by characterizing extreme (Low and High) WMC groups using a 20% and 80% (top) or using a 50% and 50% (bottom) percentile grouping. Regardless of 20/80 and 50/50 percentile grouping, results showed high WMC had larger drift-rates (V) than low WMC (i.e., main effect of group), but redundancy gain (RT vs. ST) and loss (RD vs. SD) did not depend on WMC individual differences (i.e., no group by redundancy condition interaction). Critically, both analyses show high EWM had larger drift-rates (V) than low EWM (i.e., main effect of group), but redundancy gain (RT vs. ST) and loss (RD vs. SD) effects did not depend on EWM capacity individual differences (i.e., no interaction between group and redundancy effects).

## 4. Discussion

The main findings of the present study were twofold. First, working memory visual-search processes were found to be both facilitated and inhibited under a novel redundant memory probes (RMP) task using accuracy, RT, and LBA measures of “how much” (i.e., capacity) and “how fast” (i.e., efficiency) information is processed. Second, although individuals classified as having high or low WMC with traditional dual span tasks differed in accuracy, RT, and rates of evidence accumulation on the RMP task, groups did not differ in the magnitude of facilitation (redundancy gain) and inhibition (redundancy loss) effects observed under the RMP task. When taken together, these results suggest redundant information reliably facilitates and inhibits the efficiency or speed of working memory visual search, and these effects are independent of more general limits and individual differences in the capacity or space of working memory.

### 4.1. Redundancy effects on working memory visual search

Consistent with previous research, results showed that memory probes with redundant-target features significantly improved or facilitated the accuracy and mean RT of working memory visual search relative to memory probes with only one target feature (i.e., redundancy gain). In contrast, results showed that memory probes with redundant-distractor features significantly reduced or inhibited the accuracy and mean RT of working memory visual search relative to memory probes with only one distractor feature (i.e., redundancy loss). Similarly, inhibition effects also were found for memory probes with mixed target and distractor features relative to memory probes with one distractor feature. These results also were confirmed with an LBA model of decision-making accuracy and RT that implicitly assumed a coactive mental architecture with fixed-capacity drove the rate or efficiency in which internal evidence accumulates (drift-rates) during working memory visual search. For this model, drift-rates were (i) larger (facilitated) for redundant target probes than for single target probes, (ii) smaller (inhibited) for redundant distractor probes than for single distractor probes, and (iii) smaller (inhibited) for mixed target and distractor probes than for single target probes.

In the context of Eidels et al. (2010)'s findings, the current evidence of redundancy gains in LBA model drift-rates suggest that the RMP task facilitated participant's workload efficiency to that of “super-capacity,” such that increases in the amount of to-be-processed target information lead to an increase in the rate at which evidence accumulated during working memory visual-search process. This interpretation of the current findings is inconsistent with the dominant conceptualization of working memory processes being limited capacity in nature (Baddeley, 2000). Crucially, the expectation for limited capacity would be that of inhibition or a decrease in workload efficiency, such that redundant target conditions lead to reduced accuracy, RT, and drift-rates relative to single target conditions. Therefore, the limited-capacity assumption did not hold in the present study, because evidence of “super capacity” processing was found via significant redundancy gain effects. However, the limited-capacity assumption did hold under distractor probe conditions, such that accuracy, RT, and drift-rates where impeded when contrasting (i) redundant-distractor vs. single-distractor conditions, and (ii) mixed target/distractor conditions vs. single-target conditions (see Figure 6).

One explanation for the present findings could be that the locus of working memory limited capacity is specific to short-term memory processes, and not necessarily divided-attention processes. That is, perhaps domain-specific short-term memory space is limited in capacity and can hold only a certain amount of contents, while controlled divided-attention speed is not limited in efficiency or workload capacity and can be facilitated or inhibited by the stimulus-context. Toward this end, a key limitation of the present research was that we did not take into account variability in performance as a function of variability in memory-set size (i.e., Factor 1). Specifically, RMP task memory lists were either 2, 4, or 6 items long, and thus, it could be that facilitation and inhibition effects on workload capacity during working memory visual search depend on memory list or set size. Future work with the RMP task should attempt to disentangle the interactive effects of memory set size (short-term memory) and memory probe redundancy (divided-attention search).

Another possible explanation for the present finding of “super capacity” processing under redundant-target conditions is that these effects were simply an artifact of implicitly selecting a fixed-capacity coactive process as a baseline for our LBA model. Perhaps fitting an LBA model that assumed a more conservative UCIP baseline would not yield evidence of facilitation. Therefore, the present findings are limited by questions concerning LBA model specification, and the exact configuration of mental processes driving performance in the RMP task. Future work with the RMP task might attempt to identify the best fitting baseline model at the individual subjects level, and/or use the standard UCIP model to determine the extent to which model derived differences in workload capacity (i.e., super, unlimited, or limited capacity classifications) correspond with differences in WMC on dual span tasks.

### 4.2. Working memory capacity effects on working memory visual search

Consistent with previous research, results showed that individuals classified as high WMC on traditional dual span tasks had generally more accurate and faster RMP task performance than those classified as low WMC. These results also were confirmed with the LBA model of performance that indicated higher WMC was associated with higher drift-rates. Evidence of a link between WMC and RMP task drift-rates is consistent with previous research demonstrating that WMC individual differences are predicted by drift-rates obtained under other simple reaction time tasks (Schmemiedek et al., 2007). Our findings also could be interpreted to suggest that capacity and efficiency measurements of working memory processing could stem from the same underlying source of individual differences, such that greater working memory “capacity” or processing “space” is associated with greater working memory “efficiency” or processing “speed.”

However, our results also suggest an important caveat in that redundancy gain and loss effects were not dependent on WMC. Specifically, both high and low WMC individuals showed comparable redundancy gains (facilitation) and losses (inhibition) effects in the RMP task. In fact, low and high WMC groups showed comparable evidence of “super-capacity” processing for redundant targets and “limited capacity” processing for mixed and redundant-distractors. This could be interpreted to mean that the efficiency with which individuals integrate information in working memory (i.e., workload capacity) may not depend on individual differences in working memory capacity or space limitations. However, it is important to point out that our sample recruitment and extreme groups approach may limit the generalizability of the present findings. Mainly, the use of a dichotomized WMC variable and categorical analysis (i.e., repeated measures) method limited the statistically power of the current results. Perhaps other dimensional or factor analytic methods might reveal an interaction between WMC individual differences and redundancy effects. However, it is suspected that any potential interaction effects revealed by dimensional or factor analytic approaches would be weak at best, given that the current analyses did not reveal statistical trends in favor of rejecting the null hypothesis of an interaction between WMC differences and redundancy effects.

Finally, limitations in analytic approach notwithstanding, the results of the current study have broader implications for clinical research, because working memory impairments are known to characterize individuals with a history of substance use and antisocial behavior (Finn et al., 2009; Endres et al., 2011, 2014). Current results using the extreme group approach revealed that individuals with low WMC showed poorer RMP task performance than those with high WMC. Indeed, these effects could be largely due to clinical problems, given that individuals with low WMC also tend to have a greater history of chronic, severe, and co-occurring substance abuse and antisocial behavior than those with high WMC. In this regard, another study limitation was that participants were recruited based on individual differences in clinical history, but such individual differences were not included as covariates in repeated measures analyses. Perhaps redundancy gain and loss effects are more or less apparent in those with a history of substance use and antisocial behavior. This has important clinical implications because, to the extent that the RMP task could be used to disentangle the interaction between working memory subsystems, it would be interesting to know whether the source of working memory impairments stems from deficits in divided attention, short-term memory, or both. To our knowledge, research has yet to identify the exact psychological processes and mechanisms driving working memory impairments in substance use and antisocial behavior. It is also unclear whether individuals with such conditions are more or less sensitive to redundancy information in working memory tasks. Such knowledge and specificity could provide valuable information to emerging treatment models for substance use and antisocial behavior problems that utilize working memory training or remediation as a means to improve self-regulation and impulse control. Future research with the RMP task should examine the effects of individual differences in externalizing disorders on performance, and attempt to uncover the latent psychological mechanisms driving the known working memory impairments associated with this condition.

### 4.3. Linear ballistic accumulator model of the redundant memory probes task

Lastly, results from the current study added to the growing body of research applying quantitative modeling approaches to the study of individual differences (Neufeld et al., 2002; Yechiam et al., 2005; Johnson et al., 2010; Endres et al., 2011, 2014). Here, evidence showed that measures of performance accuracy and RT we not always sensitive to differences in RMP task condition and dual span task related WMC. Specifically, for the 3 possible RMP task effects: RT vs. ST, RD vs. SD, and TD vs. ST, the accuracy (percent correct) measure detected 2 of 3, the RT (mean) measure detected 2 of 3, and the LBA drift-rates (accuracy adjusted) measure detected 3 of 3. For the 3 group effects that were possible for each RMP task effect, the accuracy (percent correct) measure detected 2 of 3, the RT (mean) measure detected 0 of 3, and the LBA drift-rates (accuracy adjusted) measure detected 3 of 3. There were no significant interaction effects between task and group for any of the 3 contrasts. These comparisons could be interpreted to mean that LBA model drift-rates were more psychometrically reliable than accuracy and RT measures, showing the greatest sensitivity to task and group main effects, while being equally selective at ruling out task by group interactions. However, it is important to note that a key limitation with the current LBA model was its specification. Specifically, we implicitly assumed that a fixed-capacity, coactive mental architecture drove visual search processes for all subjects, rather than taking steps to identify exactly which mental architecture was driving visual-search processes in the RMP task. Future quantitative modeling work should investigate this issue of model specification and identify whether RMP visual search is best represented by a coactive, parallel or serial mental architecture.

### Conflict of interest statement

The Guest Associate Editor Cheng-Ta Yang declares that, despite having collaborated with author Joseph W Houpt, the review process was handled objectively and no conflict of interest exists. The authors declare that the research was conducted in the absence of any commercial or financial relationships that could be construed as a potential conflict of interest.
